# Highly Tm^3+^ doped germanate glass and its single mode fiber for 2.0 μm laser

**DOI:** 10.1038/srep20344

**Published:** 2016-02-01

**Authors:** Xin Wen, Guowu Tang, Qi Yang, Xiaodong Chen, Qi Qian, Qinyuan Zhang, Zhongmin Yang

**Affiliations:** 1 State Key Laboratory of Luminescent Materials and Devices and Institute of Optical Communication Materials, South China University of Technology, Guangzhou 510640, P. R. China; 2 Guangdong Engineering Technology Research and Development Center of Special Optical Fiber Materials and Devices, South China University of Technology, Guangzhou 510640, P. R. China; 3 Guangdong Provincial Key Laboratory of Fiber Laser Materials and Applied Techniques, South China University of Technology, Guangzhou 510640, P. R. China

## Abstract

Highly Tm^3+^ doped optical fibers are urgently desirable for 2.0 μm compact single-frequency fiber laser and high-repetition-rate mode-locked fiber laser. Here, we systematically investigated the optical parameters, energy transfer processes and thermal properties of Tm^3+^ doped barium gallo-germanate (BGG) glasses. Highly Tm^3+^ doped BGG glass single mode (SM) fibers were fabricated by the rod-in-tube technique. The Tm^3+^ doping concentration reaches 7.6 × 10^20^ ions/cm^3^, being the reported highest level in Tm^3+^ doped BGG SM fibers. Using ultra short (1.6 cm) as-drawn highly Tm^3+^ doped BGG SM fiber, a single-frequency fiber laser at 1.95 μm has been demonstrated with a maximum output power of 35 mW when in-band pumped by a home-made 1568 nm fiber laser. Additionally, a multilongitudinal-mode fiber laser at 1.95 μm has also been achieved in a 10 cm long as-drawn active fiber, yielding a maximum laser output power of 165 mW and a slope efficiency of 17%. The results confirm that the as-drawn highly Tm^3+^ doped BGG SM fibers are promising in applications that require high gain and high power from a short piece of active optical fiber.

Fiber lasers near 2.0 μm region have been a research topic with an increasing emphasis due to the important applications such as eye-safe light detection and ranging (LIDAR), remote sensing, laser surgery, spectroscopy and mid-infrared (mid-IR) frequency generation^1^,^2^,^3^,^4^. Among the rare earth ions, Tm^3+^ and Ho^3+^ are commonly utilized to generate 2.0 μm lasers owing to Tm^3+^: ^3^F_4_→^3^H_6_ and Ho^3+^: ^5^I_7_→^5^I_8_ transition, respectively. However, due to the lack of an appropriate ground absorption band in commercial laser diode (LD), the development of Ho^3+^ doped fiber lasers are still in initial stages^5^. Tm^3+^ has gained significant attention because of its inherent advantages. It can be directly pumped by commercial 808 nm LD through ^3^H_6_→^3^H_4_ transition. Under such pump scheme, quantum efficiency of Tm^3+^ is expected to reach 200% from the cross relaxation energy transfer (^3^H_4_ + ^3^H_6_→^3^F_4_ + ^3^F_4_)^6^. In addition, the broad emission about 300 nm of Tm^3+^ allows a wide wavelength tuning range and makes it suitable for femtosecond pulse generation^7^.

It is well-known that the Tm^3+^ high doping concentration in fiber enables efficient one-for-two energy transfer by bringing the Tm^3+^ ions closer, and remarkably enhances the slope efficiency and output power of fiber lasers^8^. Moreover, the high Tm^3+^ doping concentration leads to large pump absorption and high gain per unit length of the fiber, which can reduce the required fiber length in fiber lasers and contributes to background loss reduction, nonlinear effect mitigation and narrow emission linewidth. Hence, using heavily Tm^3+^ doped fibers, small-sized short cavity single-frequency fiber lasers with narrow laser linewidth or high-repetition-rate mode-locked fiber lasers with high stability can be built. Additionally, it is possible to scale the output power of 2.0 μm fiber lasers with maintained good spatial mode qualities. Fabricating highly Tm^3+^ doped single-mode (SM) fibers has now been a hot subject in the field of 2.0 μm fiber lasers.

To obtain heavily Tm^3+^ doped fibers, the choice of the host glass is important. A high doping concentration is not achievable in commercially utilized silica glass due to its defined glass structure. The multi-component oxide glasses (e.g., silicate glass, tellurite glass, germanate glass) which allow higher Tm^3+^ solubility triggered great interest. Recently, a silicate glass fiber with high Tm_2_O_3_ doping concentration of 7 wt% (8.35 × 10^20 ^ions/cm^3^) was reported^9^. However, silicate glasses are not the ideal host glasses for mid-IR lasers since the high phonon energy could lead to fast multiphoton relaxation which decreases the quantum efficiency and also causes thermal damage of the fiber laser. 2.0 μm fiber lasers have also been realized in tellurite glass fibers, but the reported Tm_2_O_3_ doping concentration is relatively low, moreover, the low laser damage threshold and brittleness of tellurite glass fibers are still bottlenecks^10^. Germanate glasses, especially barium gallo-germanate (BGG), offers an ideal alternative, as it combines the attributes of high rare-earth solubility, comparatively low phonon energy, superior IR transparency, high damage threshold and strong mechanical strength^11^,^12^,^13^. According to the previous researches, the highest Tm_2_O_3_ doping concentration in BGG SM glass fibers is only 1 mol%^14^, thereafter; further effort is greatly needed to put into the fabrication of BGG glass SM fibers with higher Tm_2_O_3_ doping concentration.

In this work, the optical parameters, energy transfer processes and thermal properties of Tm^3+^ doped BGG glasses were systematically studied. BGG glass SM fibers with high Tm_2_O_3_ doping concentration of 1.8 mol% (7.6 × 10^20 ^ions/cm^3^) were fabricated. An in-band pumped single-frequency fiber laser operating at 1.95 μm has been realized using ultra short (1.6 cm) highly Tm^3+^ doped BGG SM fiber. Additionally, a multilongitudinal-mode fiber laser with 165 mW laser output and 17% slope efficiency has been achieved in a 10 cm long as-drawn active fiber.

## Experiments and Measurements

BGG glasses with molar composition of 20BaO-(16.8-*x*)Ga_2_O_3_-60GeO_2_-3.2(La_2_O_3_+ Y_2_O_3_)-*x*Tm_2_O_3_ (*x* = 0.2, 0.6, 1.0, 1.4, 1.8, 2.2), denoted as BGG-*x*, were prepared by the conventional melting-quenching technique using high purity reagents (99.99% minimum). For each glass, well-mixed raw materials (80 g) were melted at 1350 °C for 30 min in a covered crucible, and then dried O_2_ was bubbled into the melt for 30 min to remove the OH^−^. Subsequently, the melt was cast into a preheated steel mold and annealed before it was cooled to room temperature. The annealed samples were cut and polished for optical property measurements.

For fiber fabrication, core glass batch with the same components of BGG-1.8 and cladding glass with the molar composition of 21BaO-15Ga_2_O_3_-60GeO_2_-4(La_2_O_3_ +Y_2_O_3_), in quantity of 650 g powder, were fabricated, respectively. The melt procedure was similar to that applied to BGG-*x*, however, with longer time considering the large mass. The mixed batches were preheated at 200 °C for 3 h in a vacuum drier to remove the free water. Then, they were melted in a covered platinum crucible at 1350 °C for 1 h with the protective atmosphere of dry O_2_, after which dry O_2_ was incorporated into the melts for 40 min to eliminate the OH^−^ content at 1380 °C. The stir effect caused by bubbles could homogenize the melt and reduce the cluster of the melt^15^. To remove bubbles and stripes, the melts were stirred with a platinum rod and then clarified under dry O_2_ atmosphere. Thereafter, the melts were cast into the preheated steel mold and annealed. The rod-in-tube technique was carried out for fiber fabrication with a preform designed for the SM optical fiber, as we described in our previous work^14^. Continuous highly Tm^3+^ doped SM fibers were drawn inside the drawing tower under N_2_ controlled atmosphere.

The density was tested by Archimedes’ liquid-immersion method in distilled water. The refractive index was recorded on a prism coupling apparatus (Metricon Model 2010). The characteristic temperatures were analyzed using a Netzsch STA 449C Jupiter differential scanning calorimeter (DSC) under N_2_ atmosphere at a heating rate of 10 °C/min from 25 to 980 °C. The infrared transmittance spectra were measured using a Vector-33 FTIR spectrometer (Bruker, Switzerland). The Raman spectra were performed on a Raman spectrometer (Renishaw inVia) with a 532 nm laser as the excitation source. The absorption spectra were employed on a Perkin-Elmer Lambda 900 UV-Vis-NIR double beam spectrophotometer (Waltham, MA). Upon excitation of an 808 nm LD, the fluorescence spectra of the bulk glass samples were measured by a computer controlled Triax 320 type spectro-fluorimeter (Jobin-Yvon Corp.) with a lock-in amplifier. All the measurements were carried out at room temperature with the same experiment conditions so as to get comparable results.

## Results and Discussions

### Transmittance and Raman spectrum

**Figure 1:**
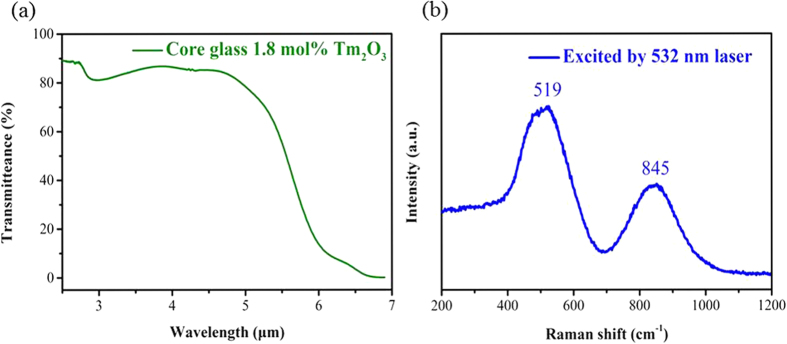
(**a**) Transmittance spectrum and (**b**) Raman spectrum of the core glass.

The OH^−^ impurities play a role of quenching centers in the energy transfer processes of Tm^3+^ ions, and it is required to remove OH^−^ content in glasses so as to achieve excellent emission property. Figure 1(a) presents the transmittance spectrum of core glass from 2.5 to 7.5 μm. As can be seen, the absorption cut-off edge is up to 6.5 μm. The broad absorption near 3 μm is corresponding to the stretching vibration of free OH^−^ groups, and the glass absorption coefficient, *α*_*OH*_ (cm^−1^) can be calculated using the following equation (1)^16^:


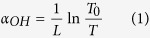


where *L* is the thickness of the sample, *T* is the transmission at 3000 nm, *T*_*0*_ is the transmission at 2600 nm. The OH^−^ absorption coefficient of core glass is 0.39 cm^−1^, lower than that of Tm^3+^ doped lead silicate glass (1.7 cm^−1^)^17^, and Tm^3+^ doped tellurite glass (0.5 cm^−1^)^18^.

The Raman technique was utilized to analyze the structure of BGG glass, and the vibrational spectrum of core glass was shown in Fig. 1(b). Two broad bands centered at 519 cm^−1^ and 845 cm^−1^ are observed which are assigned to the symmetric bending and stretching mode contributions from Ge–O–Ge or Ga–O–Ga bridging oxygen’s vibrations, as well as the asymmetric stretching vibrations of Ge–O or Ga–O structural units, respectively^19^,^20^. Meanwhile, it can be found that the largest phonon energy only extends to 845 cm^−1^, much lower than that of silicate glass (1100 cm^−1^)^21^, and lanthanum tungsten tellurite glass (920 cm^−1^)^22^. The lower phonon energy, as well as smaller OH^−^ absorption coefficient, could reduce the probability of non-radiative relaxation and thus be very conducive to Tm^3+^ 1.8 μm luminescence.

### Absorption and emission spectra

**Figure 2:**
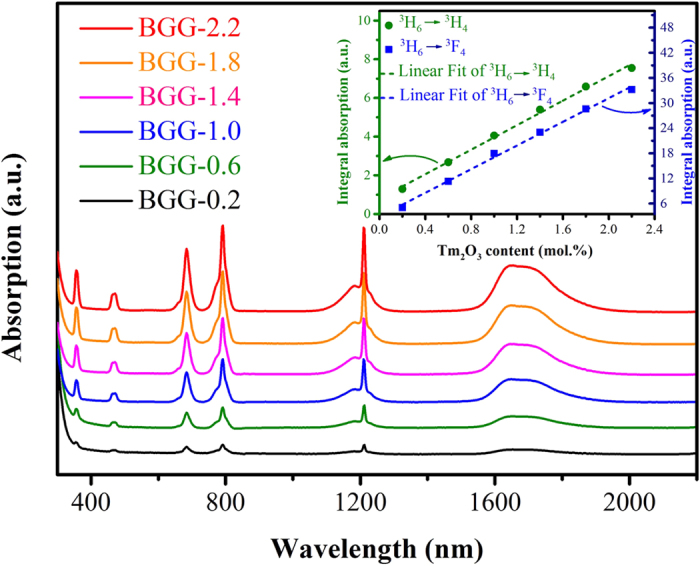
Absorption spectra of BGG glasses with different Tm_2_O_3_ doping concentration. The inset shows variation of integral absorption intensities of ^3^H_6_→^3^H_4_ and ^3^H_6_→^3^F_4_ transitions as a function of Tm_2_O_3_ doping content.

The BGG glasses with different Tm_2_O_3_ doping concentration were completely transparent and homogeneous in appearance. Figure 2 depicts the absorption spectra of these glass samples in the wavelength region of 300–2200 nm. Six characteristic absorption bands located at 1652 nm, 1210 nm, 790 nm, 660 nm and 682 nm, 468 nm, and 356 nm can be ascribed to the typical transitions from ^3^H_6_ ground state to excited state ^3^F_4_, ^3^H_5_, ^3^H_4_, ^3^F_2_ and ^3^F_3_, ^1^G_4_ and ^1^D_2_ of Tm^3+^, respectively. The obvious absorption bands around 790 nm and 1652 nm indicate that commercially available AlGaAs LD or a ~1.5 μm fiber laser can be utilized as the pump sources. As can be seen, there is no obvious change in the position of the Tm^3+^ absorption peaks, but the intensity enhances with the increment of Tm_2_O_3_ content. The solubility of rare earth ions can be estimated roughly by the variation of the integral absorption intensities with Tm_2_O_3_ content^12^,^23^. The inset of Fig. 2 shows variation of integral absorption intensities of ^3^H_6_→^3^H_4_ and ^3^H_6_→^3^F_4_ transitions as a function of Tm_2_O_3_ content. It is noted that the good linearity fittings reveal excellent solubility of Tm^3+^ in the current BGG glass system.

**Figure 3:**
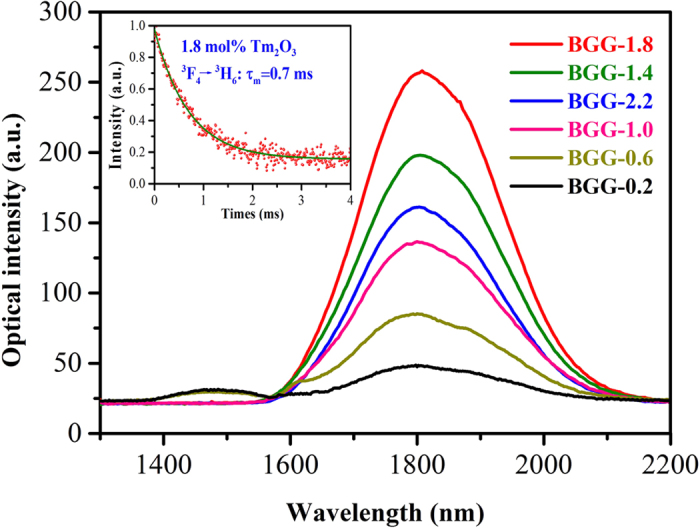
Emission spectra of BGG-*x* (*x* = 0.2, 0.6, 1.0, 1.4, 1.8, 2.2) samples upon excitation of 808 nm LD. The inset of Fig. 3 shows fluorescence decay curve of 1.8 μm emission in the core glass.

Figure 3 shows the emission spectra from 1300 to 2200 nm of BGG-*x* (*x* = 0.2, 0.6, 1.0, 1.4, 1.8, 2.2) samples upon excitation of an 808 nm LD. As can be seen, the 1470 nm emission from ^3^H_4_→^3^F_4_ transition is only observed in BGG-0.2 and BGG-0.6 samples. With increment of Tm_2_O_3_ content, the 1470 nm emission becomes negligible, and the 1.8 μm emission from ^3^F_4_→^3^H_6_ transition strengthens gradually. This phenomenon can be accounted for shorting the distance between Tm^3+^ ions which increases the cross-relaxation energy transfer (^3^H_4_ + ^3^H_6_→^3^F_4_ + ^3^F_4_) probability before it attains the maximum value at 1.8 mol% Tm_2_O_3_. The result that the 1.8 μm emission intensity decreases with further addition of Tm_2_O_3_ content may be explained by the enhanced energy transfer rate from ^3^F_4_ to OH^−^ or impurities. The inset of Fig. 3 shows the lifetime of Tm^3+^:^3^F_4_ level in the core glass. It was measured to be 0.7 ms, which is larger than that in 1.44 wt% Tm_2_O_3_ silica fiber (420 μs)^24^ and 1.46 wt% Tm_2_O_3_ silicate fiber (460 μs)^21^. The higher lifetime is favorable for population accumulation so as to achieve lasers.

It should be noted that the optimal Tm_2_O_3_ doping concentration in the current BGG system reaches 1.8 mol % (equal to 5.1 wt% of Tm_2_O_3_ and Tm^3+^: 7.6 × 10^20 ^ions/cm^3^), which is remarkably higher than that of tellurite glass (Tm^3+^: 3.76 × 10^20 ^ions/cm^3^)^25^ and lead silicate glass (1 mol%)^17^, comparable to that of other germanate glass (5 wt%)^26^, just a little lower than the reported highest Tm^3+^ doping concentration (8.35 × 10^20 ^ions/cm^3^) from silicate glass^27^. This highly Tm^3+^ doped BGG glass will be promising for some special applications such as 2.0 μm single-frequency fiber lasers and high-repetition-rate mode-locked fiber lasers.

### Cross sections and gain properties

In order to further estimate the spectroscopic properties of 1.8 μm emission in highly Tm^3+^ doped BGG glass, absorption and emission cross sections are calculated. According to the absorption spectrum, the absorption cross-section (*σ*_*a*_) can be calculated by using the Beer-Lambert law^28^:


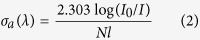


where log(*I*_*0*_*/I*) is absorptivity from absorption spectrum, *N* is the Tm^3+^ doping concentration, and *l* is the sample thickness.

Furthermore, the emission cross section (*σ*_*e*_) can be obtained from the fluorescence spectrum by using Fuchtbauer–Ladenburg (FL) equation^29^:


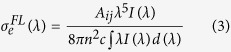


where *A*_*ij*_ is the spontaneous emission probability of the transition, *I*(*λ*) is the emission intensity, *c* is the speed of light, *n* is the refractive index, and *λ* is the wavelength.

**Figure 4:**
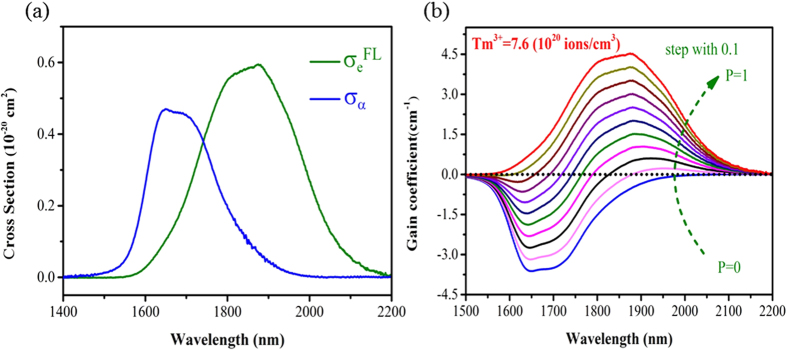
(**a**) Absorption and emission cross sections and (**b**) the calculated gain coefficient of the core glass.

Figure 4(a) shows the calculated absorption and emission cross sections of the core glass. It is found that the maximum values of *σ*_*a*_ and *σ*_*e*_^*FL*^ are 4.7 × 10^−21 ^cm^2^ at 1650 nm and 5.95 × 10^−20 ^cm^2^ at 1875 nm, respectively. The prepared core glass has higher emission cross section than that of Tm^3+^ doped ZBLAN glass (2.4 × 10^−21 ^cm^2^)^30^ and silicate glass (3.89 × 10^−21 ^cm^2^)^31^.

Based on the calculated *σ*_*a*_(λ) and *σ*_*e*_(λ), it is valuable to compute the wavelength dependence of net gain as a function of population inversion for the upper laser level so as to acquire the gain property quantitatively. Assuming that Tm^3+^ ions are either in the ground state (^3^H_6_) or in the upper laser level (^3^F_4_), the net gain coefficient, *G*(*λ*), can be expressed by the following equation^32^:


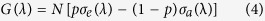


where *p* represents the population of the upper laser level, and *N* stands for the total Tm^3+^ concentration.

The calculated gain coefficient of core glass with *p* ranging from 0 to 1 with the step of 0.1 is shown in Fig. 4(b). It is found that the gain becomes positive when *p* is more than 0.1, indicating that a low pumping threshold will be required for 2.0 μm laser operation in the highly Tm^3+^ doped BGG optical fibers. In addition, the maximum gain coefficient reaches 4.53 cm^−1^ around 1875 nm due to the high Tm^3+^ doping concentration. It is three times than that of Tm^3+^ doped silicate glass (1.5 cm^−1^)^21^, showing promising applications of building compact and efficient 2.0 μm fiber lasers.

### Energy transfer microparameters

**Figure 5:**
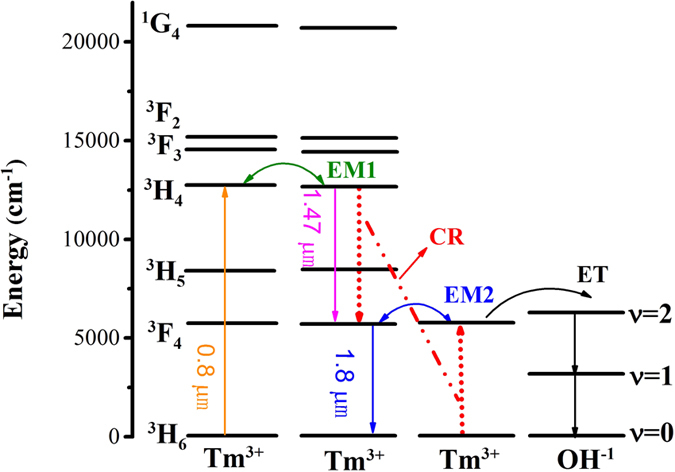
Simplified energy level scheme of Tm^3+^ pumped by an 808 nm LD.

To gain better understanding about the energy transfer processes, the energy-level diagram obtained from the absorption spectra of Tm^3+^ doped BGG glass is demonstrated in Fig. 5. The cross-relaxation transfer process (CR, ^3^H_4_ + ^3^H_6_→^3^F_4_ + ^3^F_4_), energy migration into ^3^H_4_ level (EM1, ^3^H_4_ + ^3^H_6_→^3^H_6_ + ^3^H_4_), energy migration into ^3^F_4_ level (EM2, ^3^F_4_ + ^3^H_6_→^3^H_6_ + ^3^F_4_), and energy transfer (ET) from Tm^3+^ to OH^−^ are indicated. The extended integral method is widely used to analyze energy transfer processes between the donor and acceptor. Concerning a dipole–dipole interaction, the microscopic energy transfer probability between donor (D) and acceptor (A) ions can be expressed as follows^33^:


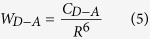


where *R* is the distance between the donor and the acceptor, *C*_*D−A*_ is the transfer constant defined as^34^:


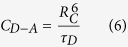


where *R*_*C*_ represents the critical radius of the interaction and *τ*_*D*_stands for the intrinsic lifetime at the donor excited level. When phonons participate in the considered process, the transfer constant can be obtained by the following equation^33^,^34^,^35^:


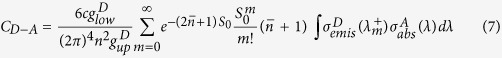


where *c* is the light speed, *n* is the refractive index of the glass, 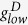
and
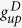
are the degeneracies of the lower and upper levels of the donor, respectively, *ħω*_*0*_ is the maximum phonon energy, 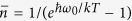 is the average occupancy of the phonon mode at temperature *T*, *S*_*0*_ is the Huang–Rhys factor (0.31 for Tm (ref. 35)), *m* is the number of the phonons that participate in the energy transfer, and 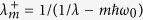 is the wavelength with m phonon creation.

According to equation (6) and equation (7), the energy migration rate between ^3^H_4_ energy levels (EM1: ^3^H_4_ + ^3^H_6_→^3^H_6_ + ^3^H_4_) and cross-relaxation (CR: ^3^H_4_ + ^3^H_6_→^3^F_4_ + ^3^F_4_) rate in BGG-0.6 sample are analyzed, as listed in Table 1. It is found that the transfer constant for energy migration process (*C*_*D–D*_) is evidently larger than that for cross-relaxation process (*C*_*D–A*_). Hence, the condition for applying the hopping model to calculate the energy transfer rate *W*_*ET*_ is fulfilled. The macroscopic CR probability, *W*_*ET*_, can be expressed by the product of transfer constants^36^:


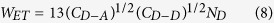


where *N*_*D*_ is the concentration of donors, and in this case the donor is Tm^3+^. According to equation (8), the *W*_*ET*_ for BGG-0.6 is calculated to 1799 × 10^−20 ^cm^3^/s, which is far larger than that of Tm^3+^ doped silicate glasses (103.4 × 10^−20 ^cm^3^/s)^34^. The results suggest that favorable cross-relaxation can be realized in the Tm^3+^ doped BGG glasses.

According to equation (8), the macroscopic CR probability *W*_*ET*_ is directly proportional to the Tm^3+^ doping concentration, which has also been proven in the previous experiment^37^, accordingly; the emission intensity at 1.8 μm should increase with Tm_2_O_3_ doping content. However, the emission results in Fig. 3 do not show this tendency, this result can be attributed to the concentration quenching phenomenon. Although Tm^3+^ shows excellent solubility in the current BGG glass, it is of great importance to select suitable Tm_2_O_3_ doping concentration to achieve optimal 1.8 μm emission.

### Thermal properties and refractive index

**Figure 6:**
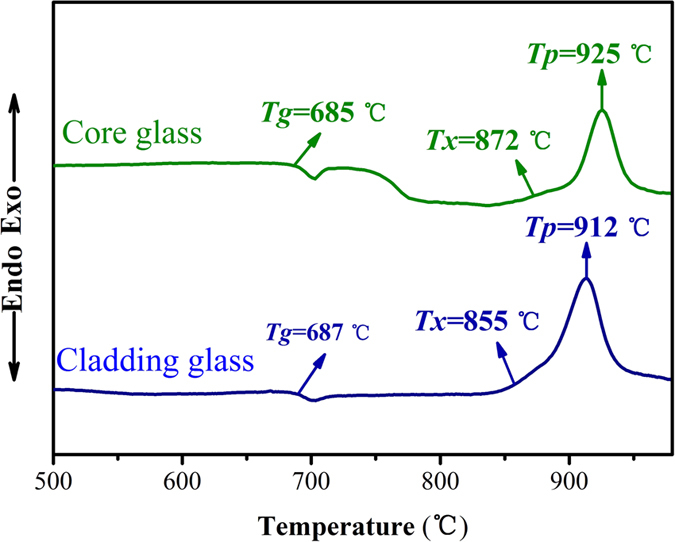
DSC curves of BGG core and cladding glasses.

Except excellent emission property, thermal stability is also a critical property for laser glasses, particularly for high rare earth doped glasses since they are easier to result in occurrence of crystallization during the fiber drawing reheating processes. The glass transition temperature (*T*_*g*_), onset crystallization peak temperature (*T*_*x*_), and the top crystallization temperature (*T*_*p*_) of the core and cladding glasses can be determined from the DSC curves, as shown in Fig. 6. The criterion, *ΔT* = *T*_*x*_−*T*_*g*_, is frequently used as an important parameter to evaluate glass thermal stability^38^,^39^. The larger the value of *ΔT* is, the better anti-crystallization ability gets. The *ΔT* of core and cladding glasses are 187 °C and 168 °C, respectively, which are far larger than that of fluorogermanate glass (144 °C)^12^, tellurite glass (102 °C)^40^ and fluorophosphate glass (87 °C)^41^. The large values indicate that BGG core and cladding glasses can possess a lower crystal nucleation and growth rate during the reheating process, which makes it possible to obtain crystal-free glass fibers. Furthermore, it should be noted that the *T*_*g*_ value of the glasses reaches 685 °C, suggesting that BGG glass possesses high ability against thermal damage^19^.

The coefficients of thermal expansion (CTE) value of core and cladding glasses were also measured in the 30–700 °C, and the values were 6.3 × 10^−6^/°C and 6.8 × 10^−6^/°C, respectively. The refractive indices of core and cladding glasses at 1533 nm are 1.735 and 1.730, respectively. It can be seen that characteristic temperatures, the CTE values and the refractive indices of core and cladding glasses are well-matched and suitable for fiber fabrication.

### Fiber laser performances

Table 2 summarizes some important basic parameters of the core and cladding glasses. It is noted that the Tm^3+^ doping concentration reaches 7.6 × 10^20 ^ions/cm^3^, being the reported highest level in Tm^3+^ doped BGG fibers to our best knowledge. The fiber preform was designed for the requirement of SM optical fibers. Continuous highly Tm^3+^ doped BGG glass fibers were successfully fabricated in-house with cladding diameter of 125 μm and core diameter of 9.2 μm by the typical rod-in-tube technique. The numerical apertures (N.A.) of the BGG glass fiber is 0.132. The propagation loss of the as-drawn Tm^3+^ BGG glass fibers at 1310 nm was measured to be 0.095 dB/cm by the cutback method, which is lower than that of tellurium germanate glass (0.15 dB/cm)^42^, but little larger than that of Tm^3+^ doped lead silicate glass fiber (0.07 dB/cm)^17^. The large fiber loss may be attributed to the impurities which were brought into the glass during the melting process.

As is well known, when the normalized frequency *V* of a fiber is less than or equal to 2.405, only the fundamental mode (LP_01_) can be propagated in the active fiber and the single-mode operation can occur. The *V* of a fiber is given as:


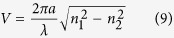


where *λ* is the wavelength, *a* is the radius of the fiber, *n*_*1*_ and *n*_*2*_ are refractive index of core and cladding glass, respectively. Assuming that *V* is equal to 2.405, the cut-off wavelength (*λ*_*c*_) for the as-drawn fiber is calculated to be 1.59 μm, suggesting that once the operation wavelength is larger than 1.59 μm, the condition for single-mode operation is satisfied. The wavelength of the Tm^3+^ fiber laser is usually around 1.8 μm, giving a result of *V* < 2.405 which indicates that the highly Tm^3+^ doped germanate fiber can be single-mode operated.

**Figure 7:**
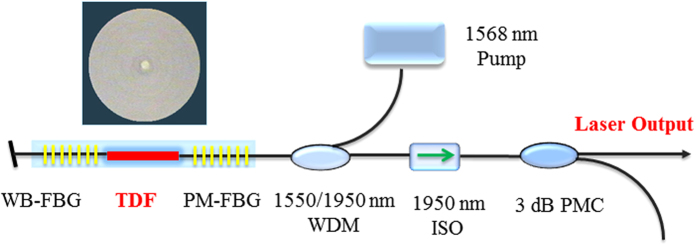
Schematic diagram of the experimental set-up for highly Tm^3+^ doped BGG fiber laser. The inset of Fig. 7 shows the photomicrograph of highly Tm^3+^ doped BGG fiber.

Figure 7 shows schematic diagram of the experimental set-up for highly Tm^3+^ doped BGG fiber laser. The inset of Fig. 7 shows the photomicrograph of highly Tm^3+^ doped BGG fiber. The laser cavity was constructed by a piece of as-drawn highly Tm^3+^ doped BGG SM fiber and a pair of spectrally narrow 1950 nm Fiber Bragg Gratings (FBGs). The FBGs have a core diameter of 7 μm and cladding diameter of 125 μm with N.A. of 0.2. The fiber laser cavity was packed into an aluminum tube, the temperature of which was strictly controlled by the thermo-electric cooler (TEC) with a resolution of 0.05 °C. A homemade watt-class 1568 nm fiber laser was utilized to in-band pump the laser cavity through a fuse-based wavelength division multiplexer (WDM) for 1550/1950 nm. The laser spectrum was recorded by an optical spectrum analyzer (OSA, Yokogawa AQ6375) with a wavelength resolution of 0.05 nm. An optical power meter (OPM, Field Mate Area Meter) with a power resolution of 0.1 mW was used to measure the laser output the power.

**Figure 8:**
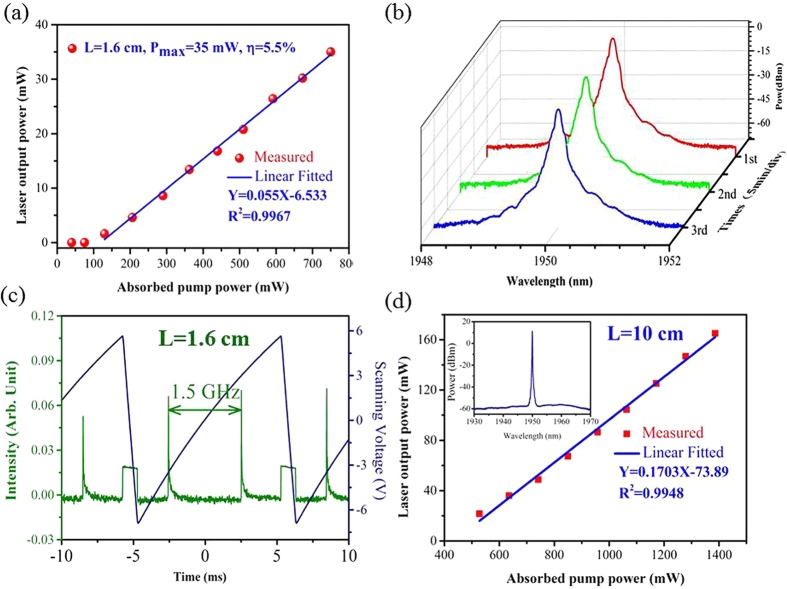
The laser output power as a function of the absorbed pump power (**a**), the laser spectra (**b**), and the scanning spectrum over FSR of the FFPI (**c**) of the fiber laser using 1.6-cm-long highly Tm^3+^ doped BGG SM fiber; (**d**) laser output power as a function of the absorbed pump power with active fiber of 10 cm; the inset of Fig. 8 (d) shows the spectrum of fiber laser using the 10-cm-long highly Tm^3+^ doped BGG SM fiber.

A fiber laser has been built and characterized using a 1.6-cm-long as-drawn highly Tm^3+^ doped BGG SM fiber as the active fiber. The effective length of the resonator includes the 1.6-cm-long highly Tm^3+^ doped germanate fiber and half of the 10-mm-long PM-FBG and WB-FBG. It is less than 2.6 cm, giving a longitudinal mode spacing >3.2 GHz. The PM-FBG utilized in the experiment has a reflection bandwidth of less than 4.7 GHz. Therefore, with a proper temperature control, the laser will operate in a single longitudinal mode without mode hop and mode competition phenomena. Figure 8(a) shows the laser output as a function of the absorbed pump power. About 28% of the launched pump power was not absorbed due to the ultra-short length of the gain fiber. The laser threshold pump power is about 130 mW, and the fiber laser yields a maximum laser output power of 35 mW with a slope efficiency of 5.5%. The laser spectra from 1948 to 1952 nm were measured three times with a time interval of 5 min, which are shown in Fig. 8(b). It can be seen that there are no obvious changes in spectrum shape and the center wavelength of the laser. The center wavelength of the laser is near 1950.02 nm, and the signal-to-noise ratio (SNR) is higher than 65 dB. Single-frequency operation of the laser was confirmed by using the scanning fiber Fabry-Perot interferometer (FFPI, SA200-18B) with free spectral range (FSR) of 1.5 GHz and resolution of 7.5 MHz. The scanning spectrum over a FSR of the FFPI, shown in the Fig. 8(c), indicates that the laser was operating in single longitudinal mode as we expected.

Additionally, an all-fiber laser performance operating in multilongitudinal-mode has also been achieved in a 10 cm long as-drawn active fiber. The launched pump power was totally absorbed due to the long length of the active glass fiber. It can be seen from the inset of Fig. 8(d) that the fiber laser output is centered at 1950.01 nm, well matches the FBG center. The laser threshold pump power is about 250 mW, and the output laser power rises linearly versus the absorbed pump power, yielding a maximum value of 165 mW and a slope efficiency of 17%, as shown in Fig. 8(d). Much higher output power could be obtained by further increasing the launched pump power, which is beyond the limitation of the handing power of the components in the cavity.

Table 3 summarizes the active fiber length, pumping wavelength, laser threshold, maximum laser output power, and slope efficiency of various fiber lasers. Compared with silica and tellurite glass fibers, a 35 mW single-frequency laser output can be obtained from the ultra-short heavily Tm^3+^ doped BGG SM fiber (1.6 cm) which confirms its high gain per unit length. Moreover, the slope efficiency from 10 cm long active fiber is twice as the result from 9.7 cm long 1 mol% Tm_2_O_3_ doped germanate glass fiber^14^, indicating that enhancing the Tm_2_O_3_ doping content in BGG glass SM fiber can lead to higher gain and higher absorption coefficient per unit length. Much higher slope efficiency and laser output of the lasers are expected by optimizing the core diameter and N.A. value of FBGs to match the as-drawn glass fibers, reducing the fiber loss and using efficient 790 nm single-mode laser pump source. The results suggest that the as-drawn highly Tm^3+^ doped BGG SM glass fibers are excellent active medium for 2.0 μm fiber lasers.

## Conclusions

The optical parameters, energy transfer processes and thermal properties of Tm^3+^ doped BGG glasses were analyzed in detail. Highly Tm^3+^ doped BGG glass SM fibers with core diameter of 9.2 μm and cladding diameter of 125 μm were designed and fabricated by the rod-in-tube technique. The Tm_2_O_3_ doping concentration reaches 1.8 mol % (Tm^3+^: 7.6 × 10^20 ^ions/cm^3^), which owns the record high Tm^3+^ doping concentration in BGG glass fibers to the best of our knowledge. A single-frequency fiber laser at 1.95 μm has been demonstrated in a 1.6 cm as-drawn highly Tm^3+^ doped BGG SM fiber with a maximum output power of 35 mW when in-band pumped by a home-made 1568 nm fiber laser. In addition, a multilongitudinal-mode fiber laser has been achieved using a 10 cm long as-drawn active fiber, corresponding to a maximum laser output power of 165 mW and a slope efficiency of 17%. The results confirm that the as-drawn highly Tm^3+^ doped BGG glass SM fiber possesses high pump absorption and high gain per unit length, which make it a promising active medium for compact and efficient 2.0 μm fiber laser.

## Additional Information

**How to cite this article**: Wen, X. *et al.* Highly Tm^3+^ doped germanate glass and its single mode fiber for 2.0 µm laser. *Sci. Rep.* **6**, 20344; doi: 10.1038/srep20344 (2016).

## Figures and Tables

**Table 1:** Transfer constant of energy migration (EM1) and cross-relaxation (CR) processes of BGG-0.6 sample.

Energy transfer	N (No. of phonons) (%phonons)	Transfer constant (×10^−39 ^cm^6^/s)	Rc (nm)	W_ET_ (10^−20 ^cm^3^/s)
Tm→Tm (CR)	0, 1, 2	C_D–A_ = 3.63	1.14	1799
^3^H_4_ + ^3^H_6_→^3^F_4_ + ^3^F_4_	3.81, 95.51, 0.68			
Tm→Tm (EM1)	0, 1	C_D–D_ = 8.16	1.29	
^3^H_4_ + ^3^H_6_→^3^H_6_ + ^3^H_4_	99.38, 0.62			

**Table 2:** The important basic parameters of the core and cladding glasses.

Glasses	Tm^3+^ concentration (10^20 ^ions/cm^3^)	*T*_*g*_ (°C)	*T*_*x*_ (°C)	*ΔT*(°C)	Refractive index (*n*)	CTE (10^−6^/°C)	N.A.
Core	7.6	685	872	187	1.735	6.3	0.132
Cladding	–	687	855	168	1.730	6.8	

**Table 3:** Summary of the active fiber length, pumping wavelength, laser threshold, maximum laser output power, and slope efficiency of various fiber lasers.

Active fiber	Fiber length (cm)	Pumping wavelength (nm)	Laser threshold (mW)	Maximum output power (mW)	Slope efficiency	Reference
Silica fiber	~5.0	790	59	1	0.2	43
Tellurite fiber	2.0	1590	260	5	~3%	44
Siliate fiber	2.0	1575	300	~40	37%	45
Germanate fiber	9.6	1568	140	140	7.6%	14
BGG fiber	10	1568	250	165	17%	This work
BGG fiber	1.6	1568	130	35	5.5%	This work
